# Lifestyle changes, mental health, and health-related quality of life in children aged 6–7 years before and during the COVID-19 pandemic in South Germany

**DOI:** 10.1186/s13034-022-00454-1

**Published:** 2022-03-11

**Authors:** Deborah Kurz, Stefanie Braig, Jon Genuneit, Dietrich Rothenbacher

**Affiliations:** 1 Institute of Epidemiology and Medical Biometry, Ulm University, Helmholtzstrasse 22, 89081 Ulm, Germany; 2 Pediatric Epidemiology, Department of Pediatrics, Medical Faculty, Klinik Und Poliklinik Für Kinder- Und Jugendmedizin, Universität Leipzig, Liebigstraße 20a, 04103 Leipzig, Germany

**Keywords:** COVID-19, Child health, Mental health, Quality of life, Wellbeing, SDQ, Lifestyle changes

## Abstract

**Background:**

The measures against the COVID-19 pandemic are challenging for children and parents, and detrimental effects on child health are suggested especially from lock-down measures and school closings.

**Methods:**

We conducted a cross-sectional analysis using a population based longitudinal (birth-) cohort study (SPATZ study) conducted in the South of Germany. Data included all 6 or 7 year old children for whom a questionnaire was completed during first grade of school. Consequently, we were able to analyze children being in first grade before the first lockdown in Germany (≤ 15th March 2020), as well as children being in first grade during the pandemic (> 15th March 2020). We conducted descriptive statistics and estimated the associations between the two time periods, before and during the pandemic, and various outcomes of child health using multivariable adjusted linear or logistic regression modeling. The analysis was stratified by gender.

**Results:**

Results among n = 362 children aged six or seven years showed substantially lower mean scores of health-related quality of life (difference in means: − 5.5, 95% confidence interval (CI) − 9.0, − 2.0), and higher mean scores in total emotional and behavioral difficulties (difference in means: 2.0, 95% CI 0.2, 3.8) in girls during vs. before the COVID-19 pandemic using multivariable linear regression modeling. In addition, weekly screen-time was increased in boys by 3.5 h (95% CI 0.6, 6.4). We did not find substantial differences in sleep quality, physical activity, and time spent with books, neither in boys nor in girls, however, the limited sample size has to be considered.

**Conclusion:**

Child health (and behavior) of first grade school children is possibly impacted by the COVID-19 pandemic with adverse consequences possibly differing by gender.

**Supplementary Information:**

The online version contains supplementary material available at 10.1186/s13034-022-00454-1.

## Introduction

The measures against the COVID-19 pandemic including lock-downs, school closings, contact restrictions, limited possibilities for leisure time activities, and parents working from home are challenging for children and parents. Careful assessment of lifestyle and psychological changes related to the pandemic is needed [1–4], considering the acute phases of the pandemic as well as the subsequent phase [3]. This is necessary to better understand possible undesirable consequences, mitigate potential negative effects, and improve return to normality [3].

Based on the limited findings of population-based, observational studies, which are ongoing before and throughout the pandemic, we aimed to describe selected outcomes of mental health, quality of life and lifestyle patterns in children aged six or seven years participating in the Ulm SPATZ Health Study (SPATZ) by means of cross-sectional analysis before and during the pandemic.

## Methods

### Study design, study population and ethical approval

SPATZ is a population based longitudinal (birth-) cohort study conducted in Ulm in the South of Germany (overall response 49%) which started recruitment of newborns and their mothers during hospitalization after delivery in the Department of Gynecology and Obstetrics, University Medical Center Ulm, in the year 2012 (which was the only maternity hospital in Ulm at this time). Details are described elsewhere [5]. Ethical approval was obtained from the Ethics Board of Ulm University (no. 311/11).

### Sampling procedure and sample description

**Fig. 1:**
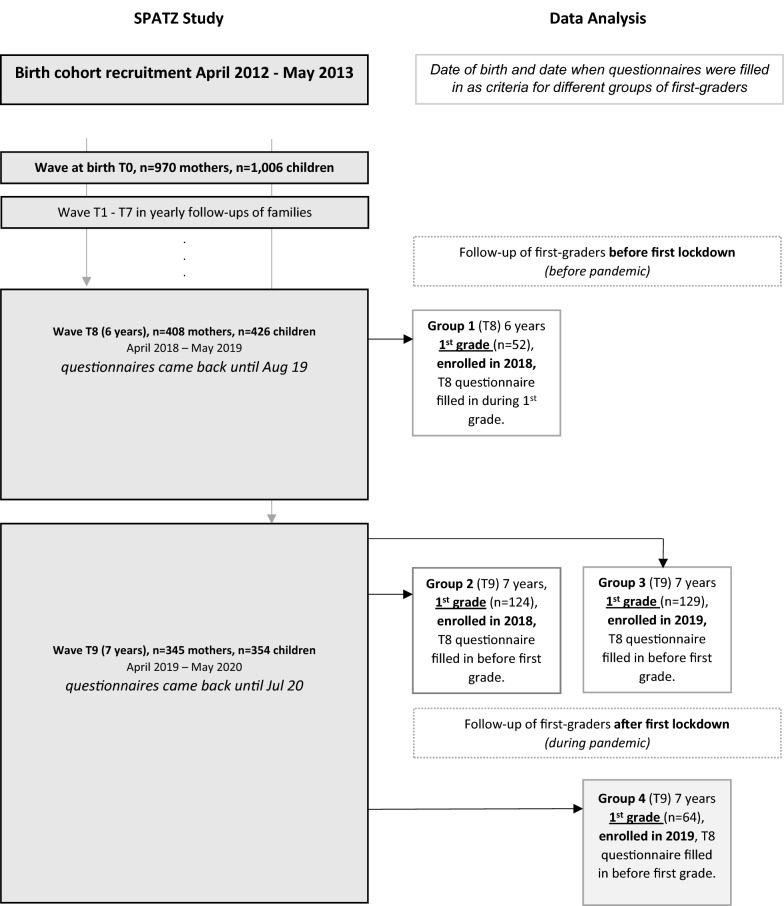
Study sample of first-graders was derived from longitudinal SPATZ cohort (birth cohort recruitment took place from April 2012 to May 2013). Four different groups of first-graders were derived (all observations were independent)

Data for this analysis included all 6 or 7 year old children of the SPATZ study (waves T8 and T9) for whom a questionnaire was completed during first grade of school. We were able to analyze in a cross-sectional manner children being in first grade before the first lockdown in Germany (≤ 15th March 2020), as well as children being in first grade during the pandemic (> 15th March 2020) (notably, schools closed at 16th March 2020). Figure 1 shows how the study sample of first-grade school-children (so called “first-graders”) was derived from the longitudinal SPATZ cohort study. In detail, four different groups of first-graders were created which differ in (1) the time of school enrollment, in (2) the follow-up waves, and in (3) the time when questionnaires were completed (before vs. during pandemic). In detail, first the study sample was divided by year of school enrollment based on date of birth. The reason was that the initial recruitment for the birth cohort at baseline was a continuous process stretched over 13 months (i.e. from April 2012 to May 2013). In a second step, we have chosen either the questionnaire of the 8th or the 9th follow-up wave. If the questionnaire of the 8th wave was completed before school enrollment of the child, data from the 9th wave was analyzed (groups 2, 3, and 4). (Only for group 1 the questionnaire of the 8th wave was completed during first grade of school, and therefore used.) A further difference between the groups 1, 2, and 3 and group 4 is that data of group 4 was gathered during the pandemic, data of all the other groups before the pandemic. For further explanation, see Fig. 1. All observations in this analysis are independent; only one time-point of assessment is included for each child.

### Measures and outcome variables

The main outcome variables of interest were parental reports of children’s health-related quality of life (German version of the KINDL-R questionnaire [6, 7]; 24 items summed up to the total score, subscales of six dimensions with four items each; higher score = higher quality of life), and emotional and behavioral difficulties (German version of the Strengths and Difficulties Questionnaire (SDQ) [8]; 20 items summed up to the total difficulties score; higher score = more difficulties). Further outcome variables were physical activity (Bayer [9]; five items for the binary classification, seven items for the score; higher score = more physical active), screen-time (items covering time spent with TV/DVD (also via computer/smartphone), time spent with computer games/game consoles (also via smartphone), time spent with other use of internet/computer (also via smartphone), data were average hours on school days and on weekends, respectively, and time spent with books (either read by themselves or read to them by someone else). The last outcome variable of interest was sleep quality (German version of the Children’s Sleep Habits Questionnaire (CSHQ) [10]; higher score = lower sleep quality). All used questionnaires are validated, except for screen-time, and time spent with books. Though, categories were similar to those used in a large German population-based study (KIGGS) [11].

### Analytical strategy

We conducted descriptive statistics and estimated the associations between the two time periods, before and during the pandemic, and various outcomes of child health using multivariable linear or logistic adjusted regression modeling. E.g., linear regression modeling was used to estimate differences in means between participants before the pandemic vs. during the pandemic, adjusted for covariates. If, in this analysis, the 95% CIs include the 0 (i.e. the so called “null-effect value”) no statistical differences in means were found. For the binary outcome logistic regression modelling was used to estimate the pandemic-related probability (odds ratio) for the presence of a given outcome vs. its absence. In this case, if the 95% CIs include the “1”, no statistical differences were found in the outcome associated to the pandemic. In comparison to linear regression were the null-effect values is 0 (see above), in logistic regression the so called “null-effect value” is 1. The analysis was stratified by gender and adjusted for age, and year of school enrollment (in order to account for the different groups of first-graders), and duration of school education of mother (educational attainment). The latter one was used as a proxy for socio economic status (SES). The analyses were performed using SAS® 9.4 (The SAS Institute, Cary, NC, USA).

## Results

### Descriptive results

We analyzed data of n = 362 children; Table 1 shows the descriptive results of the study sample. About a half (52.5%) of the study sample (first-graders) were girls, 85.6% were 7 years old, and most of the first-graders (93.9%) had a mother with German nationality. The majority of first-graders (70.7%) had a mother with high educational attainment (≥ 12 years). Roughly the half of first-graders were enrolled in 2018 (46.7%), and the others in 2019. Of all first-graders, 17.7% were in first grade of school during the pandemic).

**Table 1:** Characteristics of the study sample (n = 362 children)

	Male	Female	Total
N (%)	172 (47.5)	190 (52.5)	362
Age (years), N (%)			
6 years	26 (15.1)	26 (13.7)	52 (14.4)
7 years	146 (84.9)	164 (86.3)	310 (85.6)
Nationality mother, N (%)			
German	159 (92.4)	181 (95.3)	340 (93.9)
Other	13 (7.6)	9 (4.7)	22 (6.1)
Years of school education mother, N (%)			
≤ 9	9 (5.2)	4 (2.1)	13 (3.6)
10 to 11	38 (22.1)	53 (27.9)	91 (25.1)
≥ 12	123 (71.5)	133 (70.0)	256 (70.7)
Missing	2 (1.2)	–	2 (0.6)
School enrollment of child, N (%)			
2018	82 (47.7)	87 (45.8)	169 (46.7)
2019	90 (52.3)	103 (54.2)	193 (53.3)
Time of fist-grade			
First-grade during pandemic, N (%)	32 (18.6)	32 (16.8)	64 (17.7)
First-grade before pandemic, N (%)	140 (81.4)	158 (83.2)	298 (82.3)

Table 1 shows also, that maternal nationality (German/non-German), maternal educational attainment, age of child (6/7), year of school enrollment (2018/2019), and time of first-grade (before/during the pandemic) were almost equally distributed between boys and girls.

Table 2 shows the distribution of the before mentioned variables within the four different groups of first-graders. Maternal nationality (German/non-German) and children’s gender were also almost equally distributed in the different groups of first-graders. Furthermore, descriptive statistics (means and standard deviations) of all analyzed outcomes differed across the groups of first-graders and gender (Table 2).

**Table 2:** Descriptive results of several health related outcomes in n = 362 children in first grade of school, before and during COVID-19 pandemic, stratified by gender

COVID-19 pandemic	Before the pandemic (until 15th March 2020)						During the pandemic	
Group no.	1		2		3		4	
N	52		117		129		64	
Age (years)	6		7		7		7	
School enrollment	2018		2018		2019		2019	
Girls, N (%)	26 (50%)		61 (52.1%)		71 (55%)		32 (50%)	
Nationality mother, N (%)								
German	50 (96.1)		111 (94.9)		122 (94.6)		57 (89.1)	
Other	2 (3.9)		6 (5.1)		7 (5.4)		7 (10.9)	
Years of school education mother, N (%)								
≤ 9	2 (3.9)		3 (2.6)		3 (2.3)		5 (7.8)	
10 to 11	10 (19.2)		35 (29.9)		28 (21.7)		18 (28.1)	
≥ 12	39 (75.0)		79 (67.5)		98 (76.0)		40 (62.5)	
missing	1 (1.9)		–		–		1 (1.6)	
*Characteristics of child health*	N		N		N		N	
Health-related quality of life^a^, mean (SD)								
Girls	23	79.5 (9.1)	57	85.1 (8.1)	71	86.3 (7.1)	27	80.8 (8.6)
Boys	23	73.6 (11.7)	53	82.8 (6.4)	57	83.6 (10.5)	29	82.1 (9.0)
School								
Girls	23	66.3 (8.2)	58	90.9 (11.3)	71	91.7 (9.0)	28	85.3 (12.8)
Boys	24	63.8 (11.5)	53	88.0 (8.9)	57	88.3 (14.0)	30	89.4 (9.5)
Social contacts								
Girls	23	77.7 (15.5)	60	83.8 (11.3)	71	82.5 (10.1)	28	76.6 (16.5)
Boys	23	73.9 (18.3)	56	78.3 (13.0)	58	78.7 (14.1)	30	74.4 (21.1)
Family								
Girls	23	83.2 (9.7)	61	83.4 (10.6)	71	85.7 (9.2)	32	79.4 (10.7)
Boys	24	76.6 (17.0)	56	82.3 (10.2)	58	82.0 (12.1)	32	79.3 (12.6)
Self-esteem								
Girls	23	81.0 (12.7)	61	80.5 (12.7)	71	81.3 (11.1)	30	74.8 (14.4)
Boys	23	73.4 (17.8)	56	76.5 (10.5)	58	77.8 (15.8)	32	74.8 (14.9)
Physical well-being								
Girls	23	83.7 (12.0)	61	85.3 (13.0)	71	89.5 (11.1)	29	85.3 (9.8)
Boys	24	75.8 (15.9)	56	86.3 (12.0)	58	87.9 (10.7)	32	88.9 (9.5)
Emotional well-being								
Girls	23	85.3 (11.1)	61	85.6 (11.0)	71	86.8 (10.1)	30	84.2 (8.9)
Boys	24	79.7 (14.5)	56	85.3 (13.4)	58	85.3 (13.6)	32	83.2 (12.3)
Emotional and behavioral difficulties^b^, mean (SD)								
Girls	26	7.0 (6.0)	61	6.0 (3.8)	71	5.0 (3.6)	32	7.0 (5.3)
Boys	26	10.0 (7.5)	56	7.4 (4.6)	58	6.0 (5.2)	32	7.9 (5.7)
PA-Score^c^ mean (SD)								
Girls	26	0.9 (3.1)	61	1.9 (3.0)	71	1.4 (3.1)	32	1.8 (2.4)
Boys	26	0.9 (2.7)	56	1.9 (2.9)	58	1.5 (2.9)	32	1.9 (3.3)
Physical inactive^d^, N (%)								
Girls	24	13 (54.2)	60	24 (40.0)	71	34 (47.9)	31	14 (45.2)
Boys	26	15 (57.7)	56	21 (37.5)	58	25 (43.1)	32	12 (37.5)
Screen-time, h/week^e^, mean (SD)								
Girls	25	9.9 (10.2)	61	6.2 (5.0)	70	6.6 (4.9)	31	7.4 (4.9)
Boys	26	5.2 (4.8)	56	7.3 (7.4)	57	6.3 (5.1)	31	10.2 (8.4)
Time spent with books h/week^f^, mean (SD)								
Girls	25	8.1 (5.9)	61	7.4 (5.6)	70	6.0 (3.6)	31	6.1 (3.5)
Boys	26	7.1 (5.1)	56	7.2 (4.0)	57	6.0 (4.3)	31	6.3 (5.4)
Sleep quality (CSHQ)^g^, mean (SD)								
Girls	22	45.3 (6.1)	58	44.7 (5.6)	63	45.2 (5.2)	31	46.1 (4.8)
Boys	25	48.0 (7.6)	50	45.8 (6.1)	53	45.4 (7.8)	29	44.2 (6.1)

PA, physical activity; h, hours; SD, standard deviation. Details of groups 1–4 see “Methods”

^a^KINDL questionnaire, higher values indicate higher health-related quality of life

^b^Strengths and Difficulties Questionnaire (SDQ), total difficulties score, higher values indicate more emotional and behavioral difficulties

^c^Items answered with ‘physical active’ outweighing items answered with ‘physical inactive’; Score from −7 to + 7

^d^At least three of five categories were rated with infrequent physical active

^e^Including time spent with TV/DVD (also via computer/smartphone), time spent with computer games/game consoles (also via smartphone), time spent with other use of internet/computer (also via smartphone)

^f^Either read by themselves or read to them by someone else

^g^Child Sleep Habits Questionnaire (CSHQ), higher values indicate more sleep problems

Comparing the descriptive results of the three groups before the pandemic (group 1–3), 6-year old first-graders (group 1) showed in several outcomes lower numeric scores then the 7-year old first-graders (groups 2, and 3).

The group of first-graders during pandemic (group 4) achieved lower numeric scores in almost all domains of health-related quality of life compared to same-aged first-graders before the pandemic (group 2–3). The same pattern was found for the overall SDQ-score (total difficulties score), and screen-time. The latter outcome was highest in first-graders during the pandemic (group 4) compared to same aged first-graders before the pandemic (group 2–3) (Table 2). However, descriptive results for the domain ‘physical well-being’ showed in boys higher values during the pandemic (group 4) than all groups before the pandemic (group 1, 2, 3).

### Changes in outcomes associated with the pandemic

Adjusted linear regression modelling showed statistically significant lower mean scores of health-related quality of life among girls during vs. before the COVID-19 pandemic (b: − 5.5, 95% confidence interval (CI) − 9.0, − 2.0) (Table 3). Similar results were found for emotional and behavioral difficulties: Mean total difficulties scores (SDQ) increased in girls by 2.0 points (95% CI 0.2, 3.8). There were no significant differences in physical activity, time spent with books, and sleep quality during vs. before the pandemic. Notably, the only substantial effect in boys was increased weekly screen-time by 3.5 h per week (95% CI 0.6, 6.4).

**Table 3:** Associations between the COVID-19 pandemic and several health related outcomes in n = 362 children in first grade of school, adjusted for age, year of school enrollment, educational attainment mother using linear/logistic regression models, stratified by gender (boys vs. girls)

	Health related quality of life (KINDL)^a^		Emotional and behavioral difficulties (SDQ)^b^		PA-score (−7 to +7)^c^		Physical inactive^d^		Screen-time (hours/week)^e^		Time spent with books (hours/week)^f^		Quality of sleep (CSHQ)^g^	
	Differences in means (95% CI)	p-value	Differences in means (95% CI)	p-value	Differences in means (95% CI)	p-value	Odds ratio (95% CI)	p-value	Differences in means (95% CI)	p-value	Differences in means (95% CI)	p-value	Differences in means (95% CI)	p-value
COVID-19 pand														
G	− 5.5 (− 9.0, − 2.0)	0.002	2.0 (0.2, 3.8)	0.03	0.3 (− 0.9, 1.5)	0.6	0.9 (0.4, 2.1)	0.8	0.6 (− 1.8, 2.9)	0.6	− 0.03 (− 2.0, 1.9)	0.98	0.9 (− 1.4, 3.2)	0.4
B	−1.0 (− 5.2, 3.2)	0.6	1.3 (− 1.1, 3.7)	0.3	0.6 (− 0.7, 1.9)	0.4	0.7 (0.3, 1.9)	0.5	3.5 (0.6, 6.4)	0.02	0.5 (− 1.5, 2.5)	0.6	− 1.4 (− 4.6, 1.8)	0.4
N observations														
G	N = 178		N = 190		N = 189		N = 186		N = 187		N = 187		N = 174	
B	N = 160		N = 170		N = 170		N = 170		N = 168		N = 168		N = 155	

School enrollment: 09/2018 or 09/2019

Educational attainment mother: duration of school education ≤ 9 years, 10–11 years, or ≥ 12 years

G, girls; B, boys; vs, versus; CI, confidence interval; PA, physical activity

^a^KINDL questionnaire, higher values indicate higher health-related quality of life

^b^Strengths and Difficulties Questionnaire (SDQ), total difficulties score, higher values indicate more emotional and behavioral difficulties

^c^Items answered with ‘physical active’ outweighing items answered with ‘physical inactive’; Score from −7 to +7

^d^Logistic regression model, modelling the probability for being physically inactive vs. physical active

^e^Including time spent with TV/DVD (also via computer/smartphone), time spent with computer games/game consoles (also via smartphone), time spent with other use of internet/computer (also via smartphone)

^f^Either read by themselves or read to them by someone else

^g^Child Sleep Habits Questionnaire (CSHQ), higher values indicate more sleep problems

## Discussion

Our results showed that child health (and behavior) of first grade school children is possibly impacted by the COVID-19 pandemic with adverse consequences differing by gender.

### Mental well-being

In particular, we found short-term differences in mental health in girls, whereas in boys mental health did not appear to be negatively affected in the short term (i.e. no statistical significant difference on scores of KINDL-R and SDQ); however, the limited sample size has to be considered. Our results, based on data collected with the same setting before and during the pandemic in first-graders, showing decreased mental health during the pandemic are in line with several other studies [2, 12, 13]. For example, Ravens-Sieberer et al. also found that children and adolescents experienced more mental health problems and higher anxiety levels during the COVID-19 pandemic (compared to data from a representative longitudinal cohort study conducted in Germany before the pandemic) [2]. Further results from Vogel et al., investigating data from Germany, and Luijten et al., investigating data from the Netherlands, also found decreased mental health in children and adolescents during the pandemic [12, 13].

### Gender differences

Our study showed different results for boys and girls. Girls were substantially more affected in mental health, whereas boys showed vastly increased screen-time. The indicated gender-differences in being adversely affected by the pandemic, especially the possible detrimental effects on girl’s mental health are strengthened by other findings. For example, a study conducted under boys and girls in Norway revealed that girls are mentally more affected by the pandemic and more concerned to become infected with the SARS-COV-2 virus than boys [14]. Also Zhou et al. found that in Chinese adolescents girls were at higher risk for depressive and anxiety symptoms during the pandemic [15]. Further underpinned by results from Schmidt et al. revealing female gender as a main factor to be more affected by the pandemic [16]. Hence, further evaluation of gender-specific short-term and long-term pandemic effects on mental health is highly warranted.

### Socioeconomic status and pandemic

Although expected and in contrast to previous results [2, 12, 17, 18], the adverse consequences found do not largely differ according to socioeconomic status (SES): educational attainment of the mother was (only) significantly associated with emotional and behavioral difficulties in boys (Additional file 1). A previous nationwide study from Germany revealed that children from families with low SES, limited living space, or migration background have been more affected by the pandemic [2]. Furthermore, living situations and family compositions (e.g. single-parent family, having three or more children) have also been shown to be associated with more adverse mental and social health consequences during the pandemic [13]. Our contrasting results may be explained by the smaller portion of mothers with low educational attainment in our study with limited statistical power to uncover differences across education levels.

### Media use

Our result regarding increased media use in boys by 3.5 h per week is strengthened by other evidence, which strongly indicates that screen-time substantially raised during the lockdown [12, 19, 25]. An even stronger boost was found in older children [25]. Vogel et al. found that before the pandemic media use was different between weekends and weekdays (odds ratio (OR) 3.77, p < 0.001), whereas during pandemic it was found to be not [12]. Comparing weekdays during the pandemic to weekdays before the pandemic, media use (TV/DVD/video) increased by an OR of 3.80 (p < 0.001) [12]. Meaning, TV/DVD/video consumption at weekdays/weekends during the pandemic was similar to media use at weekends before the pandemic [12]. Poulain et al. found that during the pandemic higher media use was associated with lower SES [19]. In our study, educational attainment was not associated with media use. However, the relatively low number of mothers with a low educational attainment in our study has to be considered. From a public health perspective it is of high need to further evaluate changes in the total amount of screen-time among children [26].

### Other outcomes

There were also domains where we did not find substantial differences during vs. before the pandemic neither in boys nor in girls: physical activity, quality of sleep, and time spent with books. Those findings are somewhat in discrepancy to those of Vogel et al. who found that during the pandemic physical well-being decreased in children and adolescents aged 9 to 18 years, with stronger differences in children with medium/low SES [12]. Notably, the children were considerably older compared to our study and the KIDSSCREEN questionnaire was answered by the child. Contrary, Poulain et al. found that in 0–10-year-old German children (mean age 5.56 years; range 1.44–10.69) playing outside increased while the lock-down was ongoing, regardless of SES [19]. Authors assessed two time-points during lock-down, 1 month apart, and a specific questionnaire was completed by one parent [19]. Age, different methodology, as well as possibly different public health measures depending on regional, current incidence of Sars-Cov-2 infections might have contributed to the diverging results. Notably, in Germany it was always allowed for children to go outside during daytime. However, during some periods of the pandemic, playgrounds and other recreational activities were closed. Nevertheless, the descriptive results of the domain ‘physical well-being’ of the health-related quality of life questionnaire (KINDL-R) showed for boys higher physical well-being during the pandemic compared to the three groups of first-graders before the pandemic. Those results are only descriptive, however would, if found statistically significant, underpin the results from Poulain et al. Despite the contrary evidence currently available [12, 19–21], it is of high need to further investigate changes in physical activity related to a lockdown/pandemic, as the impact of reduced physical activity and prolonged sedentary behavior is related to several negative health outcomes [20, 22, 23] as well as academic achievements [24].

There are limited studies assessing sleep quality in elementary school students during the pandemic. Findings from a survey in Italy revealed, that sleeping patterns changed in all age-groups (1–3, 4–5, 6–12, and 13–18 years) and sleep disorders increased during lockdown [25]. This study was a survey via social media during the pandemic asking retrospective questions to estimate perceived changes before and during the pandemic. A specific questionnaire was arranged for the survey including a modified version of the Sleep Disturbance Scale for children (SDSC) and completed by caregivers. Also Lokandwala et al. found changes in sleeping patterns during the pandemic [27]. This study used an Actiwatch Spectrum Plus watch to track sleeping behavior and compared it to data sampled from the same subject before the pandemic. The study population was younger than the participants in our study: mean age at pandemic follow-up 56.4 months, SD = 10.8, range: 36–70 months. This study suggested changes in overnight sleep duration (longer), and sleep-mid-point and wake onset were later. However, no differences in sleep time, and sleep onset were found. The reason for the discrepancy between our findings (no apparent changes in sleep quality) and the existing reports may lie in the different methodology used and in the limited sample size of our study, leading to a possible lack of power to demonstrate a difference. However, it has to be noted that the determinants of children’s sleep behavior are scarcely understood, with moderate evidence for screen-time [4, 28].

Consequently, it is important to recognize the need of fit-for-purpose measurements and measures increasing child health (i) during the long phase out of the pandemic, (ii) during future pandemics, as well as (iii) to be prepared for future pandemics, as it was shown that pre-pandemic behaviors predict during-pandemic behaviors [29]. Whether gender-specific measures are indicated requires further assessment and corroboration in larger samples.

## Study limitations and strengths

Our study has limitations: Interpretation of our results was limited by sample size. In addition, we had a high proportion of families with high educational attainment of mother at study entry which is representative for the local population, but in families with low education and migration background loss to follow up was higher, especially during the first year of follow-up. Also, the fact that assessing the impact of COVID-19 pandemic on child health was not an a-priori hypothesis of the Ulm SPATZ Health Study, has to be taken into account. In addition, we lacked specifically generated data on possible effects of the COVID-19 pandemic, meaning we only had the routinely collected study data and no special pandemic related questionnaires which were assessed cross-sectional. Those limitations prevented us from exploring causality and behavioral patterns relating to the short-term impact of the COVID-19 pandemic on child health. On the other hand, using routinely assessed data could be a strength of the longitudinal SPATZ study, as it is not an intended COVID-19 pandemic-related study, hence preventing several forms of bias, arising from selection and awareness in participants. Meaning, especially for possible pandemic-effects, selection bias, recall bias, and conscious bias, respectively cognitive bias can be minimized when routinely assessed data is used.

### Future directions

Further investigations could account for previous/existing mental health problems in children and parents, as it was shown that especially vulnerable groups suffer more under the pandemic and that parent’s mental health during the pandemic was related to the child-SDQ-scores [30]. This could shed light on the intended hypothesis, that supporting parent’s mental health might also be helpful for protecting children’s mental health. Further analyses should also take data for living-situations and family compositions into account.

Since we analyzed cross-sectional all first grade school children in the SPATZ study, a further longitudinal investigation (taking the above mentioned data into account) is highly warranted. Hence, trajectories of three different points in time (e.g. three waves of a longitudinal cohort study of which one wave would be during the pandemic) could bring more clarity about the possible causal effects of the COVID-19 pandemic on children’s mental health and lifestyle changes.

## Conclusions

Despite the above-mentioned limitations we conclude that the health (and behavior) of first grade school children may be impacted by the COVID-19 pandemic with adverse consequences possibly differing by gender. Particularly, health-related quality of life, and emotional and behavioral difficulties were worse in girls, and screen-time was higher in boys during vs. before the pandemic.

## Supplementary Information

**Additional file 1: **Associations between the COVID-19 pandemic and the covariates used in the linear and logistic regression models in n = 362 children in first grade of school, stratified by gender (boys vs. girls).

## Data Availability

The datasets generated during and/or analyzed during the current study are not publicly available due to ethical restrictions regarding data protection issues and the study-specific consent text and procedure, but anonymized data are available from the corresponding author on reasonable request. Supplemental results on all variables included in the multivariable regression analysis are available upon request.

## Abbreviations

SPATZ: Ulm SPATZ Health Study
OR: Odds ratio
CI: Confidence interval
SD: Standard deviation
PA: Physical activity
SDQ: Strengths and Difficulties Questionnaire
CSHQ: Child Sleep Habits Questionnaire
Vs: Versus
SES: Socio-economic-status
H: Hours
