# Supplementary material for: Lifestyle changes, mental health, and health-related quality of life in children aged 6–7 years before and during the COVID-19 pandemic in South Germany
Source: Child Adolesc Psychiatry Ment Health. 2022 Mar 11;16:20. doi: 10.1186/s13034-022-00454-1 (PMC8915143; doi:10.1186/s13034-022-00454-1)
Supplement: Supplementary file 1 — Additional file 1: Associations between the COVID-19 pandemic and the covariates used in the linear and logistic regression models in n = 362 children in first grade of school, stratified by gender (boys vs. girls). [file 13034_2022_454_MOESM1_ESM.docx]

**Additional file 1.** Associations between the COVID-19 pandemic and the covariates used in the linear and logistic regression models in n=362 children in first grade of school, stratified by gender (boys vs. girls).

|  | |  | **Health related quality of life (KINDL)^a^** | | **Emotional and behavioral problems (SDQ)^b^** | | **PA-Score**  **(-7 to +7)^c^** | | **Physical Inactive^d^** | | **Screen-time**  **(hours/week)^e^** | | **Time spent with books (hours/week)^f^** | | **Quality of Sleep (CSHQ)^g^** | |
| --- | --- | --- | --- | --- | --- | --- | --- | --- | --- | --- | --- | --- | --- | --- | --- | --- |
|  | |  | **Differences in Means**  **(95% CI)** | **p-value** | **Differences in Means**  **(95% CI)** | **p-value** | **Differences in Means**  **(95% CI)** | **p-value** | **Odds Ratio**  **(95% CI)** | **p-value** | **Differences in Means**  **(95% CI)** | **p-value** | **Differences in Means**  **(95% CI)** | **p-value** | **Differences in Means**  **(95% CI)** | **p-value** |
| **COVID-19-pandemic** | | **b** | See Table 3 | | | | | | | | | | | | | |
|  | | **g** |  |  |  |  |  |  |  |  |  |  |  |  |  |  |
| **Adjusted for:** | |  |  |  |  |  |  |  |  |  |  |  |  |  |  |  |
| Age (7 vs. 6) | | b | **8.5 (3.8, 13.1)** | **<0.001** | -2.2 (-4.7, 0.4) | 0.097 | 1.0 (-0.4, 2.4) | 0.2 | 0.4 (0.2, 1.1) | 0.09 | 1.7 (-1.4, 4.8) | 0.3 | 0.4 (-1.7, 2.6) | 0.7 | -1.4 (-4.8, 1.9) | 0.4 |
|  |  | g | **5.6 (1.8, 9.4)** | **0.004** | -1.0 (-3.0, 1.0) | 0.3 | 1.0 (-0.4, 2.3) | 0.2 | 0.6 (0.2, 1.5) | 0.2 | **-3.7 (-6.3, -1.1)** | **0.005** | -0.6 (-2.8, 1.5) | 0.6 | -0.7 (-3.3, 1.9) | 0.6 |
| Year of school enrollment (19 vs.18) | | b | 0.7 (-2.7, 4.2) | 0.7 | -1.3 (-3.3, 0.7) | 0.2 | -0.4 (-1.5, 0.7) | 0.4 | 1.3 (0.6, 2.7) | 0.5 | 0.7 (-3.1, 1.7) | 0.6 | -1.4 (-3.0, 0.3) | 0.1 | -0.3 (-3.0, 2.3) | 0.8 |
|  |  | g | 1.2 (-1.6, 3.9) | 0.4 | -1.0 (-2.5, 0.5) | 0.2 | -0.5 (-1.5, 0.5) | 0.4 | 1.4 (0.7, 2,8) | 0.4 | 0.6 (-1.3, 2.6) | 0.5 | -1.3 (-2.9, 0.3) | 0.1 | 0.5 (-1.4, 2.4) | 0.6 |
| Education mother | |  |  |  |  |  |  |  |  |  |  |  |  |  |  |  |
|  | 10-11y.vs ≤9 y. | b | 3.1 (-4.1, 10.2) | 0.7 | **-5.1 (-9.0, -1.1)** | **0.01** | 1.7 (-0.4, 3.9) | 0.1 | 0.3 (0.06, 1.5) | 0.1 | -0.7 (-5.5, 4.0) | 0.8 | 1.5 (-1.8, 4.9) | 0.4 | -3.1 (-8.2, 2.0) | 0.2 |
|  | 10-11y.vs ≤9 y. | g | 1.1 (-6.9, 9.1) | 0.8 | -2.3 (-6.7, 2.1) | 0.3 | -0.003 (-3.0,3.0) | 0.998 | 0.8 (0.1, 6.1) | 0.8 | 2.2 (-3.5, 7.9) | 0.4 | 0.9 (-3.8, 5.6) | 0.7 | -2.1 (-8.3, 4.1) | 0.5 |
|  | ≥12y. vs. ≤9 y. | b | 2.5 (-4.2, 9.1) | 0.5 | **-5.1 (-8.8, -1.4)** | **0.007** | 1.6 (-0.4, 3.6) | 0.1 | 0.4 (0.08, 1.6) | 0.2 | -2.8 (-7.3, 1.6) | 0.2 | 2.7 (-0.4, 5.8) | 0.09 | -2.7 (-7.4, 2.0) | 0.3 |
|  | ≥12y. vs. ≤9 y. | g | -0.1 (-7.9, 7.8) | 0.98 | -3.0 (-7.3, 1.3) | 0.2 | -0.6 (-3.5, 2.3) | 0.7 | 0.8 (0.1, 6.2) | 0.9 | -1.6 (-7.1, 3.9) | 0.6 | 0.1 (-4.5, 4.8) | 0.95 | -2.0 (-8.1, 4.0) | 0.5 |
|  | |  |  |  |  |  |  |  |  |  |  |  |  |  |  |  |
| N observations | | b | N=160 |  | N=170 |  | N=170 |  | N=170 |  | N=168 |  | N=168 |  | N=155 |  |
|  |  | g | N=178 |  | N=190 |  | N=189 |  | N=186 |  | N=187 |  | N=187 |  | N=174 |  |

**g=girls**; **b=boys;** vs=versus; CI=confidence interval; PA=physical activity.

^a^ KINDL questionnaire, higher values indicate higher health-related quality of life

^b^ Strengths and Difficulties Questionnaire (SDQ), total difficulties score, higher values indicate more emotional and behavioral difficulties

^c^ Items answered with ‘physical active’ outweighing items answered with ‘physical inactive’; Score from -7 to +7

^d^ Logistic regression model, modelling the probability for being physically inactive vs. physical active

^e^ Including time spent with TV/DVD (also via computer/smartphone), time spent with computer games/game consoles (also via smartphone), time spent with other use of internet/computer (also via smartphone)

^f^ Either read by themselves or read to them by someone else

^g^ Child Sleep Habits Questionnaire (CSHQ), higher values indicate more sleep problems
